# Pneumococcal Immunization Reduces Neurological and Hepatic Symptoms in a Mouse Model for Niemann-Pick Type C1 Disease

**DOI:** 10.3389/fimmu.2018.03089

**Published:** 2019-01-07

**Authors:** Tom Houben, Inês Magro dos Reis, Yvonne Oligschlaeger, Hellen Steinbusch, Marion J. J. Gijbels, Tim Hendrikx, Christoph J. Binder, David Cassiman, Marit Westerterp, Jos Prickaerts, Ronit Shiri-Sverdlov

**Affiliations:** ^1^Department of Molecular Genetics, School of Nutrition and Translational Research in Metabolism, Maastricht University, Maastricht, Netherlands; ^2^Department of Psychiatry and Neuropsychology, School for Mental Health and Neuroscience, Maastricht University, Maastricht, Netherlands; ^3^Department of Laboratory Medicine, Medical University of Vienna, Vienna, Austria; ^4^Center for Molecular Medicine, Austrian Academy of Sciences, Vienna, Austria; ^5^Liver Research Unit, University of Leuven, Leuven, Belgium; ^6^Department of Gastroenterology-Hepatology and Metabolic Center, University Hospitals Leuven, Leuven, Belgium; ^7^Section Molecular Genetics, Department of Pediatrics, University of Groningen, University Medical Center Groningen, Groningen, Netherlands

**Keywords:** Niemann-Pick type C1, oxidized low-density lipoprotein, pneumococcal immunization, inflammation, lipid metabolism

## Abstract

Niemann-Pick type C1 (NPC1) disease is caused by a deleterious mutation in the *Npc1* gene, causing lysosomal accumulation of unesterified cholesterol and sphingolipids. Consequently, NPC1 disease patients suffer from severe neurovisceral symptoms which, in the absence of effective treatments, result in premature death. NPC1 disease patients display increased plasma levels of cholesterol oxidation products such as those enriched in oxidized low-density lipoprotein (oxLDL), a pro-inflammatory mediator. While it has been shown that inflammation precedes and exacerbates symptom severity in NPC1 disease, it is unclear whether oxLDL contributes to NPC1 disease progression. In this study, we investigated the effects of increasing anti-oxLDL IgM autoantibodies on systemic and neurological symptoms in an NPC1 disease mouse model. For this purpose, *Npc1*^*nih*^ mice were immunized with heat-inactivated *S. pneumoniae*, an immunogen which elicits an IgM autoantibody-mediated immune response against oxLDL. *Npc1*^*nih*^ mice injected with heat-inactivated pneumococci displayed an improved hepatic phenotype, including liver lipid accumulation and inflammation. In addition, regression of motor skills was delayed in immunized *Npc1*^*nih*^. In line with these results, brain analyses showed an improved cerebellar phenotype and neuroinflammation in comparison with control-treated subjects. This study highlights the potential of the pneumococcal immunization as a novel therapeutical approach in NPC1 disease. Future research should investigate whether implementation of this therapy can improve life span and quality of life of NPC1 disease patients.

## Introduction

Niemann-Pick type C1 (NPC1) disease is a rare, fatal disorder caused by mutations in the *Npc1* gene which result in the accumulation of unesterified cholesterol and glycosphingolipids in late endosomes/early lysosomes (1–3). The earliest clinical findings of NPC1 disease are mostly related to systemic problems in the liver (neonatal jaundice, liver failure (4–6) and the spleen (splenomegaly and severe abdominal pain resulting in splenectomies (7), and increased sensitivity to spontaneous infections (8). While, in most patients, the systemic symptoms resolve over time, some develop severe systemic problems resulting in premature death (4). Besides the systemic manifestation of the disease, NPC1 disease is also characterized by severe disturbances in the central nervous system leading to the progressive impairment of motor and cognitive function (2). In spite of significant advances in the development of therapeutic interventions for NPC1 disease (9, 10), there is currently no curative treatment available.

While lysosomal lipid accumulation is at the root of NPC1 disease, secondary pathological mechanisms such as oxidative stress (11), apoptosis (12), and inflammation (13, 14) have been shown to accompany and even contribute to NPC1 disease progression. Similarly, atherosclerosis and non-alcoholic steatohepatitis (NASH) also feature the aforementioned disease mechanisms (15, 16) and lysosomal lipid accumulation in macrophages (17). These observations suggest a mechanistic link between the pathological mechanisms of NPC1 disease, atherosclerosis, and NASH. Of note, increasing anti-oxLDL IgM autoantibody levels in the latter metabolic diseases ameliorates the macrophage-mediated inflammatory reaction (18–20). Relevantly, multiple research papers have described the cholesterol oxidation products 7-ketocholesterol and cholestane-3β, 5α, 6β-triol, two products abundantly present in oxLDL (21, 22), as sensitive and specific blood-based biomarkers for diagnosing NPC1 disease (6, 23, 24). However, whether these cholesterol oxidation products contribute to NPC1 disease progression has to our knowledge never been investigated.

Given the mechanistic overlap between the pathologies of NPC1 disease and of the metabolic diseases atherosclerosis and NASH, the aim of the current study was to determine whether increasing anti-oxLDL IgM autoantibodies reduces NPC1 disease symptoms. For this purpose, *Npc1*^*nih*^ mice were immunized with heat-inactivated *Streptococcus pneumoniae*. *Npc1*^*nih*^ mice are a well-established NPC1 disease model in which a mutation in *Npc1* leads to NPC1 truncation and loss of function (25). This mouse model recapitulates some of NPC1 disease most prominent features, including severe lysosomal cholesterol accumulation in most organs and motor function deficits (26–30). Immunization of mice using inactivated *S. pneumoniae* has been established as an effective approach to increase serum titers of anti-oxLDL IgM autoantibodies by means of molecular mimicry (18, 31). In line with our hypothesis, immunized *Npc1*^*nih*^ mice showed reduced motor function decay over time, decreased neuro- inflammation, and degeneration, reduced inflammation in liver and spleen and an improvement in liver lipid metabolism. These findings provide evidence for the involvement of anti-oxLDL IgM autoantibodies in NPC1 disease progression and highlight vaccination strategies leading to the elevation of anti-oxLDL IgM titers as a promising therapeutic approach to reduce NPC1 disease symptoms.

## Materials and Methods

### Preparation of Immunogen

For immunization, the heat-inactivated R36A strain of *S. pneumoniae* (Birmingham, AL) was used, still bearing the PC headgroup epitope similar to oxLDL. Colonies of the R36A strain were harvested at mid-log phase after incubation at 37°C on blood agar plates and transferred to Todd-Hewitt plus 0.5% yeast broth. The mid-log phase is characterized by an optical density (OD) value of 0.425–0.45 at 600 nm. *S. pneumoniae* were heat-inactivated at 60°C for 30 min; afterwards, no colonies of this suspension were detected on blood agar plates, thus confirming their inactivation. For freezer stocks of strain R36A, small aliquots of *S. pneumoniae* at mid-log density were harvested and suspended in Todd-Hewitt plus 80% sterile glycerol and stored at −80°C (32).

### Mice and Immunization

*Npc1*^*nih*^ mice were housed under standard conditions and were given free access to food and water. A complementary experiment with age-matched wildtype mice was performed to confirm the phenotypical effect of the *Npc1* mutation in *Npc1*^*nih*^ mice. Experiments were performed according to Dutch laws and were approved by the Animal Experiment Committee of Maastricht University. *Npc1*^*nih*^ mice were derived from heterozygous founders (C57BL/6 / *Npc1*^*nih*^).

The immunization protocol started in 2-week old *Npc1*^*nih*^ mice (male or female) fed a normal chow diet. Mice were divided into 2 groups (*n* = 12 for both groups). One of the groups received the equivalent of 10^8^ colony-forming units of heat-inactivated pneumococcal immunogen emulsified in 200 μl of sterile 0.9% NaCl for the primary subcutaneous immunization; subsequently, two intraperitoneal booster immunizations were administered every 2 weeks (31). The control group received NaCl injections. During the experiment, which lasted 5 weeks in total, mice were given a normal chow diet. Blood from the tail vein was collected at week 3 and 5. An overview of the experimental set-up is given in Supplementary Figure S1. During the course of the study, one control-treated *Npc1*^*nih*^ mouse died, and was therefore excluded from the experimental analyses. All tissues were isolated and snap-frozen in liquid nitrogen and stored at −80°C or fixed in 4% formaldehyde/PBS. The collection of blood and tissue specimens, RNA isolation, cDNA synthesis and qPCR were determined as described previously (18, 33, 34). Also the measurements of the autoantibody titers against IgG and IgM antibodies to CuOx-LDL and PC-BSA have been described extensively (18).

### Lipid Measurements

Total plasma cholesterol and triglyceride levels were measured (1489232, Chol CHOD-PAP, Roche, Almere, the Netherlands; 337-B, TG GPO-trinder, Sigma Aldrich, Zwijndrecht, the Netherlands). Measurements were done according to manufacturer's protocols on a Benchmark 550 Micro-plate Reader (170-6750XTU, Bio-Rad, Veenendaal, the Netherlands). For organ lipid analyses, ~50 mg of frozen liver was homogenized for 30 s at 5,000 rpm in a closed tube with 5.0 mm glass beads and 1.0 ml SET buffer (Sucrose 250 mM, EDTA 2 mM, and Tris 10 mM) (35). Complete cell destruction was done by two freeze-thaw cycles and 3 times passing through a 27- gauge syringe needle and a final freeze-thaw cycle. Protein content was measured with the BCA method (23225, Pierce, Rockford, IL, United States). TG were measured as described above. All analyses were performed according to manufacturers' instructions.

### Immunohistochemistry

Frozen liver sections (7 μm) were fixed in acetone and blocked for endogenous peroxidase by incubation with 0.25% of 0.03% H_2_O_2_ for 5 min. Primary antibodies used were against neutrophils (rat anti-mouse Ly6C, clone NIMP-R14), hepatic macrophages (rat anti-mouse CD68, clone FA11), and infiltrated macrophages and neutrophils (rat anti-mouse Mac-1 [M1/70]). 3-Amino-9-ethylcarbazole (AEC) was applied as color substrate and hematoxylin for nuclear counterstain. Sections were enclosed with Faramount aqueous mounting medium.

Paraffin-embedded cortical and cerebellar sections (4 μm; sagitally) were deparaffinized and stained with hematoxylin-eosin (HE) and primary antibodies against Iba-1 and calbindin. Sections were subjected to heat-mediated antigen retrieval and blocking with 4% goat serum/PBS, after which they were incubated with rabbit anti-Iba1 overnight. The following day, sections were incubated with swine anti-rabbit, whose signal was amplified with the VECTASTAIN^®^ Elite^®^ ABC system and counterstained with a DAB solution. For calbindin immunostaining, sections' endogenous peroxidase activity was blocked by incubating in 0.5% H_2_O_2_/methanol for 10 min. After performing heat-mediated antigen retrieval, sections were incubated with rabbit anti-calbindin overnight. The following day, sections were incubated with biotinylated goat anti-rabbit antibody, whose signal was amplified by using the VECTASTAIN^®^ Elite^®^ ABC system.

Pictures were taken with a Nikon digital camera DMX1200 and ACT-1 v2.63 software (Nikon Instruments Europe, Amstelveen, The Netherlands).

Cerebellar Purkinje cells (calbindin) were counted and scored in 10–20 microscopical views (original magnification, 200x). Number of Purkinje cells was indicated as number of cells per pixel (Adobe Photoshop CS2 v.9.0.). Scoring was performed according to presence of Purkinje cells' dendritic structure (1, defined arborization structure; 2, intermediary; 3, lack of arborization structure). Purkinje cells' number and scoring are derived from the average of independent observations from two blinded researchers.

Cerebellar Purkinje cells' HE histological staining was assessed in 10 microscopical views (original magnification, 100x) by quantifying HE staining using Fiji plugin of ImageJ software (v. 1.52 h).

Cortical microglia (Iba-1) were counted and scored in 12 microscopical views (original magnification, 400x) and indicated as number of cells per field. Microglia were scored according to their phenotype as follows: type 1 (characterized by small cell bodies and long, thin fillaments); type 2 (thicker and less numerous fillaments); type 3 (large cell body, no fillaments) (Supplementary Figure S2) (36). Microglia numbers and scoring are given as the average of independent analyses done by two blinded researchers.

Hepatic foamy cells (HE) were assessed in five microscopical views (original magnification, 200x) by an experienced pathologist and given a score in arbitrary units (A.U). Splenic fat accumulation (HE) was evaluated in three microscopical views (original magnification, 200x) by quantifying the percentage of positive area using Adobe Photoshop CS2 v.9.0.

Hepatic neutrophils (NIMP) and infiltrated macrophages and neutrophil cells (Mac-1) were counted in six microscopical views (original magnification, 200x) and were indicated as number of cells per square millimeter (cells/mm^2^). Immunostainings for hepatic macrophages (CD68) were evaluated by an experienced pathologist and given a score in arbitrary units (A.U.).

### Genotyping

Genotypes of animals were determined by PCR analysis of tail DNA. Tails were clipped at postnatal day 6 and homogenized in DirectPCR-Tail (Peqlab, Erlangen, Germany) supplemented with a tenth part Proteinase K (Qiagen, Hilden, Germany). Three hours of incubation at 56°C and agitation at 1,000 rounds per minute on a Thermo Mixer were followed by 45 min of heating at 85°C to inactivate the proteinase. Samples were then spun at full speed in a benchtop centrifuge for 1 min. The PCR reactions were performed with 0.5 ml of the obtained extracts. Each lysate underwent two PCRs; Primers gccaagtaggcgacgact and catctactgggtctccatatgtat identified the wild-type allele and primers gccaagtaggcgacgact and ttccaattgtgatctttccaa identified the mutant allele. Both PCRs were carried out under similar cycling conditions.

### Pole Test

The pole test was performed as previously described with minor modifications (37, 38). The mouse was placed head-upward on a small platform on top of a vertical rough-surfaced pole (diameter 10 mm; height 50 cm) and the time until the mouse descended to the floor (locomotor activity time: T_LA_) was recorded with a maximum duration of 120 s. Even if the mouse descended part of the way and fell the rest of the way, the behavior was scored until it reached to the floor. When the mouse did not turn downward and instead dropped from the pole, T_LA_ was considered as 120 s because of maximal severity. The pole test was performed on 3 different days (day 18, 25, and 32), with 5 consecutive trials per mouse.

### Accelerod (Accelerated Rotarod) Test

Balance and motor coordination of mice was evaluated using accelerod performance on day 21 and day 33 of the experiment. The accelerod system for mice was used under standard room conditions. The apparatus consisted of a base platform and a rotating rod with a grooved surface. Before accelerod testing, mice were trained at 2 separate days (day 17 and 19 of the experiment). On both training days, mice underwent 3 separate training moments (separated 2 h from eachother) in which they endured 3 trials with a constant speed of 18 revolutions per minute for 120 s. When operated in the acceleration modus, the rotation increased from 4 to 40 revolutions per minute in 30 s steps within 5 min. The performance was measured as the latency to fall (s).

### Statistical Analysis

Data were statistically analyzed by performing two-tailed non-paired *t*-test, two-way ANOVA followed by Tukey's *post*-*hoc* correction and repeated measures two-way ANOVA followed by Tukey's *post*-*hoc* correction using GraphPad Prism version 6 for Windows. Data were expressed as the mean and standard error of the mean. Data were considered significantly different compared to control-treated *Npc1*^*nih*^ mice, except for the analyses of rotarod and pole test results, in which comparisons were made between different time points among the same study groups. (^*^p ≤ 0.05; ^**^p < 0.01; ^***^p < 0.001; ^****^p < 0,0001).

## Results

### *Npc1^*nih*^* Mice Recapitulate NPC1 Disease Features and Display Elevated Anti-oxLDL IgM Autoantibody Titers After Immunization With Heat-Inactivated Pneumococci

**Figure 1:**
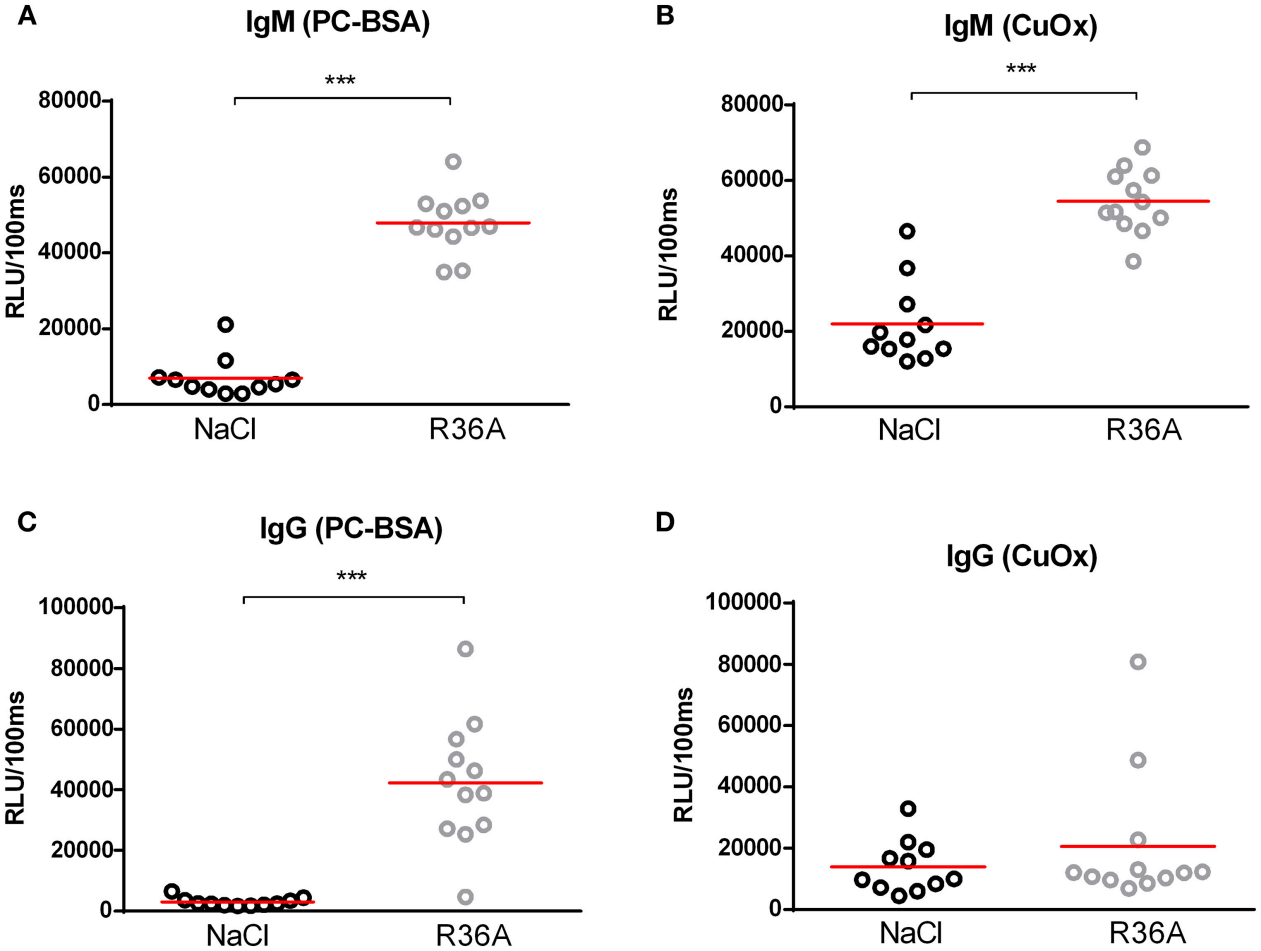
IgM autoantibodies in *Npc1*^*nih*^ mice receiving control or heat-inactivated pneumococci injections. **(A–D)** IgM and IgG antibodies against oxLDL (CuOx and PC-BSA) were measured in the plasma of control (*n* = 11) and pneumococci-immunized (*n* = 12) *Npc1*^*nih*^ mice. Data are expressed as relative light units RLU/100 ms and were triplicate determinations. ^***^Indicates *p* < 0.001 compared to NaCl-treated *Npc1*^*nih*^ mice by use of two-tailed unpaired *t-*test. *n* = 11–12 mice per group. All error bars are SEM.

To assess the effects of the *Npc1*^*nih*^ mutation, body, spleen and liver weight were compared between control-treated *Npc1*^*nih*^ mice and age-matched wildtype mice. Whereas, total spleen weight was reduced in *Npc1*^*nih*^ mice compared to wildtype mice, total liver weight was increased in *Npc1*^*nih*^ mice (Supplementary Table S1). As expected, body weight of *Npc1*^*nih*^ mice was reduced in comparison with age-matched wildtype mice (Supplementary Table S1), reflecting overall NPC1 disease burden. To determine whether anti-oxLDL IgM autoantibodies have a protective effect in NPC1, 2 week-old *Npc1*^*nih*^ mice were immunized with heat-inactivated pneumococci, known to induce high anti-oxLDL IgM titers dominated by T15-idiotypic IgM (18, 31). While total body weight (Supplementary Figure S3A) and total liver weight did not differ (Supplementary Figure S3B), total spleen weight was decreased in immunized *Npc1*^*nih*^ mice compared to control-treated counterparts (Supplementary Figure S3C). As expected, immunization resulted in a strong increase in plasma anti-oxLDL antibody levels in *Npc1*^*nih*^ mice (Figures 1A,B). Only weak IgG responses were observed, which is in line with previous reports showing pneumococcal immunizations triggering an IgM-dominated thymus-independent type-2 response highly specific for phosphorylcholine (PC) (Figures 1C,D).

### Delayed Motor Skill Degeneration and Improved Neuroinflammation and Cerebral Morphology in Immunized *Npc1^*nih*^* Mice

**Figure 2:**
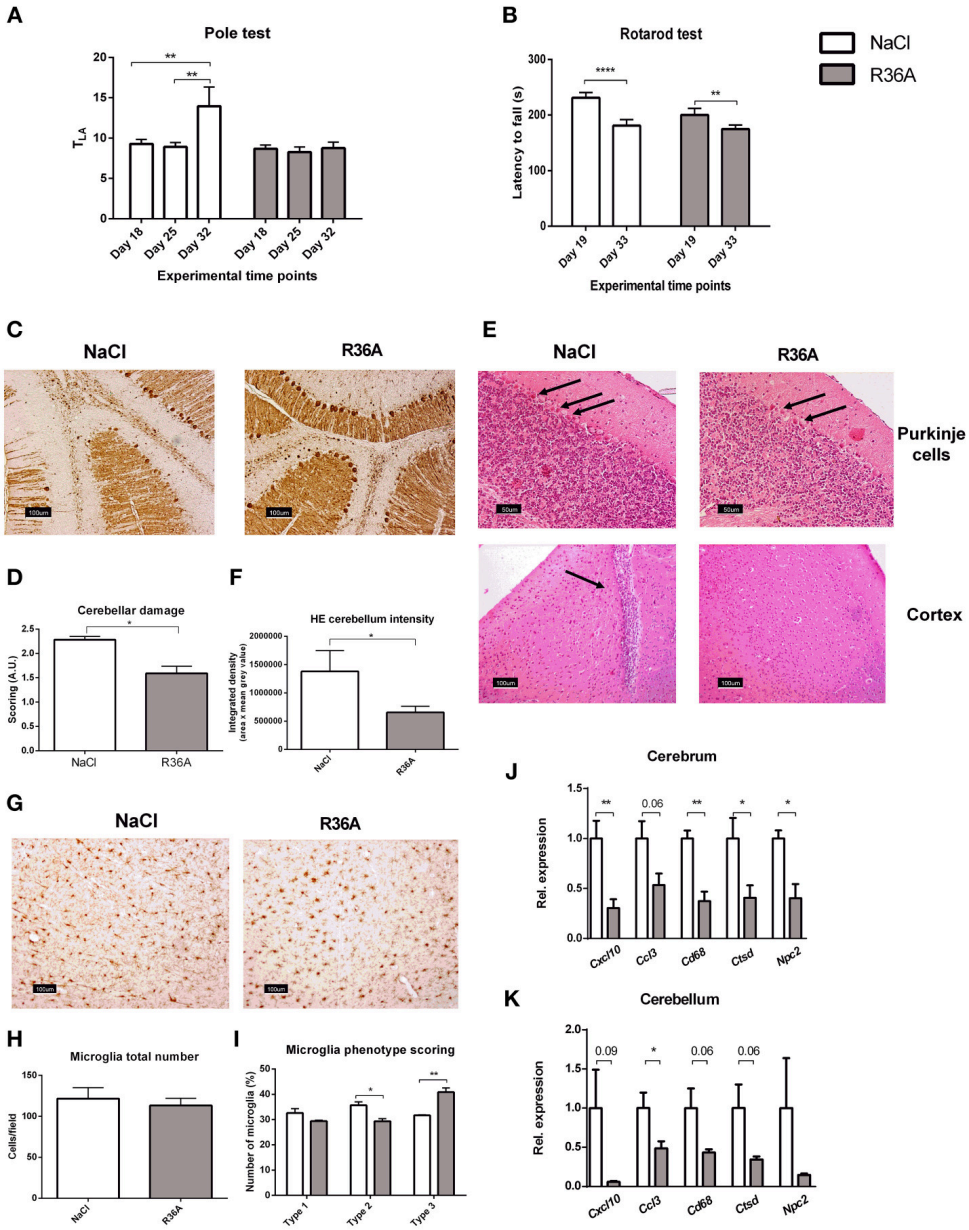
Neurological parameters. **(A)** Locomotor activity time (T_LA_) of control and immunized *Npc1*^*nih*^ mice as measured by the pole test at day 18, 25, and 32 of the experiment. **(B)** Rotarod performance of control and immunized *Npc1*^*nih*^ mice at day 19 and 33 of the experiment. **(C,D)** Representative pictures and scoring of calbindin staining of Purkinje cells in cerebellum. **(E)** General histology of the brain by HE staining. Arrows indicate Purkinje cells (*top panels*) and immune cells (*bottom left panel*). **(F)** Quantification of Purkinje HE staining density. **(G–I)** Representative pictures, quantification, and scoring of Iba-1 staining of cortical microglia (type 1, type 2, and type 3). **(J,K)** Gene expression analysis of *Cxcl10, Ccl3, Cd68, Npc2*, and *Ctsd* in cerebrum and cerebellum. Data are shown relative to NaCl-treated *Npc1*^*nih*^ mice for all analyses except for pole test and rotarod data, where comparisons were made between different time points within the same experimental group. ^*^Indicates *p* < 0.05, ^**^p < 0.01, and ^****^p < 0.0001 by use of two-tailed unpaired *t-*test or repeated measures two-way ANOVA with Tukey's *post-hoc* correction (in pole test and rotarod data analysis only). *n* = 11–12 mice per group for pole and rotarod tests and *n* = 5 mice per group for gene expression data. All error bars are SEM.

Motor skill degeneration is a common neurological feature of NPC1, due to the progressive loss of Purkinje cells. To determine the effects of the pneumococcal immunization on motor function of *Npc1*^*ni*^^h^ mice, pole and rotarod tests were performed throughout the study period. As expected, *Npc1*^*nih*^ mice performance on both tests was consistently worse than their wildtype counterparts (Supplementary Table S1), confirming NPC1 disease burden on motor function. By the end of the study, control-treated *Npc1*^*ni*^^h^ mice took significantly longer time to descend the pole (Figure 2A), whereas locomotor activity time (T_LA_) of immunized *Npc1*^*ni*^^h^ mice remained stable over the entire experiment. Additionally, the motor function scores obtained for the pole test strongly correlate with the levels of plasma anti-oxLDL IgM autoantibodies, suggesting a link between the amount of circulating anti-oxLDL IgM autoantibodies and overall motor performance (Supplementary Figure S4). While the performance of both control-treated and immunized *Npc1*^*ni*^^h^ mice on the rotarod test worsened significantly over time (Figure 2B), the observed performance decay was more prominent in the control-treated *Npc1*^*ni*^^h^ mice. These results indicate that motor function deterioration over time was decreased in immunized *Npc1*^*nih*^ mice.

Next, we investigated whether pneumococcal immunization affects Purkinje cells in *Npc1*^*nih*^ mice. In line with motor skill function results, the integrity of cerebella of control-treated *Npc1*^*n*^^ih^ was severely compromised, whereas immunized *Npc1*^*n*^^ih^ mice retained more of their cerebellar structure (Figures 2C,D and Supplementary Figure S5A). Furthermore, Purkinje cells of untreated *Npc1*^*ni*^^h^ mice displayed increased hematoxylin and eosin (HE) staining intensity, suggesting higher activation levels due to damage (Figure 2E, upper panel; Figure 2F and Supplementary Figure S5B). Overall, these results indicate that pneumococcal immunization delays cerebellar neurodegeneration in *Npc1*^*n*^^ih^ mice. In addition to severe cerebellar neurodegeneration, cortical immune cells were observed in control-treated *Npc1*^*nih*^ mice, in contrast with immunized *Npc1*^*nih*^ mice, suggesting higher levels of neuroinflammation in the control-treated group (Figure 2E, lower panel and Supplementary Figure S5B).

We further analyzed the effects of pneumococcal immunization on neuroinflammation by examining microglial cells using immunohistochemical staining for Iba-1 (Figure 2G). While no differences were observed in total number of microglia (Figure 2H), microglia of immunized *Npc1*^*nih*^ mice exhibited larger cell bodies and shorter, less ramified processes, suggesting a change in microglia phenotype following pneumococcal immunization (Figure 2I).

Finally, we analyzed cerebral and cerebellar gene expression analysis of the inflammatory markers C-X-C motif chemokine 10 (*Cxcl10*), chemokine (C-C motif) ligand 3 (*Ccl3*), and cluster of differentiation 68 (*Cd68*). Immunization of *Npc1*^*nih*^ mice reduced expression of all inflammatory markers in the cerebrum, with the exception of *Ccl3*, in which a trend suggesting decreased expression was observed (Figure 2J). In addition, while expression of *Cxcl10* and *Cd68* in the cerebellum tended to be decreased, expression of *Ccl3* was reduced in the cerebellum of immunized *Npc1*^*nih*^ mice (Figure 2K). Furthermore, gene expression of cathepsin D (*Ctsd*), a marker previously linked to NPC1 severity in mice (39, 40), and *Npc2*, which is known to bind to cholesterol in the lysosome, were reduced in the cerebrum of *Npc1*^*nih*^ mice that received the immunization, indicating an improved neurological phenotype after immunization. Notably, cerebral and cerebellar expression of several of these markers correlated with the plasma anti-oxLDL IgM autoantibody levels (Supplementary Figure S4), underlining the link between the neurological phenotype and anti-oxLDL IgM autoantibody levels. Altogether, these results indicate beneficial effects of immunization with heat-inactivated pneumococci on the neurological phenotype of *Npc1*^*nih*^ mice.

### Decreased Hepatic and Splenic Lipid Levels in Immunized *Npc1^*nih*^* Mice

**Figure 3:**
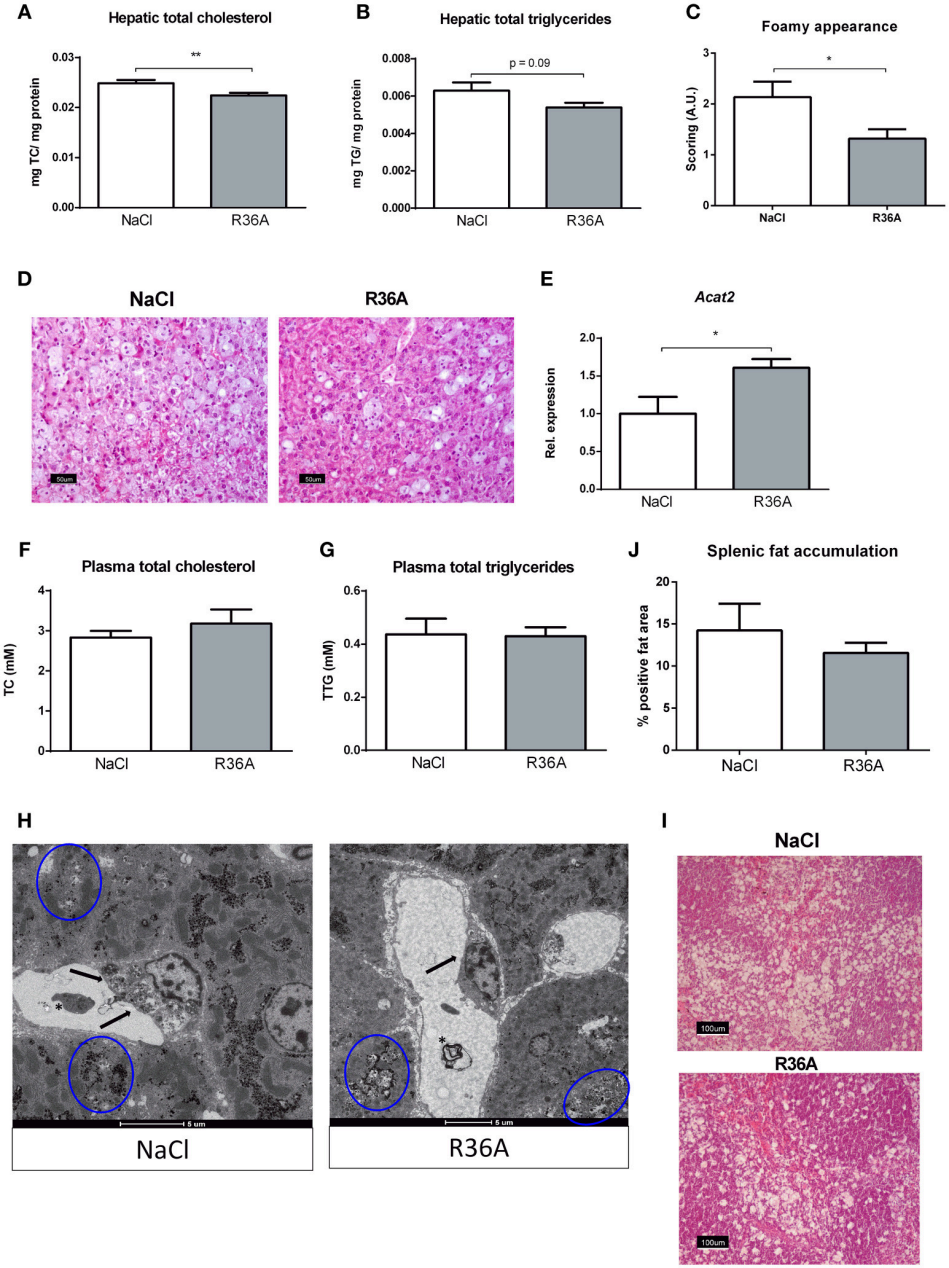
Parameters of lipid metabolism. **(A,B)** Hepatic total cholesterol and total triglyceride levels of control and immunized *Npc1*^*nih*^ mice (*n* = 11 in both experimental groups). **(C,D)** Scoring and representative pictures of hepatic HE-staining (200x magnification). **(E)** Hepatic gene expression analysis of *Acat2*. Data is shown relative to NaCl-treated *Npc1*^*nih*^ mice. **(F,G)** Plasma total cholesterol and total triglyceride levels of 7 week-old control and immunized *Npc1*^*nih*^ mice. **(H)** Electron microscopy of livers of control and immunized *Npc1*^*nih*^ mice. Arrows indicate a hepatic macrophage, blue circles indicate lipid inclusions, and asterisks indicate vacuoles with electron-dense material. **(I,J)** Representative pictures and quantification of HE staining of the spleen (100x magnification). ^*^Indicates *p* < 0.05 and ^**^p < 0.01 compared to NaCl-treated *Npc1*^*nih*^ mice by use of two-tailed unpaired *t-*test. *n* = 11–12 mice per group. All error bars are SEM.

To determine whether immunizing *Npc1*^*nih*^ mice with heat-inactivated pneumococci influences hepatic lipid levels, biochemical assessment of hepatic cholesterol and triglycerides was performed. *Npc1*^*nih*^ mice showed an increase in plasma and liver cholesterol levels compared to age matched wildtype mice (Supplementary Table S1). Seven week-old *Npc1*^*nih*^ mice that received the immunization demonstrated reduced levels of hepatic total cholesterol (Figure 3A) and triglycerides (Figure 3B) compared to control-treated *Npc1*^*nih*^ mice, though the latter only showed a trend. In line, HE-staining of the liver confirmed the decrease of lipid levels in hepatocytes and the reduced foamy appearance of hepatic macrophages in immunized *Npc1*^*nih*^ mice (Figures 3C,D). Relevantly, hepatic gene expression levels of acetyl-CoA acetyltransferase 2 (*Acat2*), the enzyme responsible for cholesterol esterification, were elevated in immunized *Npc1*^*nih*^ mice, suggesting increased cholesterol esterification upon immunization (Figure 3E). In contrast, no differences were observed in plasma lipid levels between control-treated and immunized *Npc1*^*nih*^ mice (Figures 3F,G). To obtain a more detailed view on hepatic lipid distribution, electron microscopy analysis was performed (Figure 3H). Though both control and immunized *Npc1*^*nih*^ mice showed the NPC1 characteristics of hepatic lipid inclusions (*blue circles*) and vacuoles with electron-dense material (*asterisk*), immunized *Npc1*^*nih*^ mice demonstrated a less foamy appearance of hepatic macrophages (*arrow*). In contrast with these observations, no differences were observed in lipid accumulation in the spleen of immunized *Npc1*^*nih*^ mice and control-treated *Npc1*^*nih*^ mice (Figures 3I,J). Overall, these results suggest improved hepatic lipid metabolism after increasing anti-oxLDL IgM autoantibody levels in *Npc1*^*nih*^ mice.

### Improved Hepatosplenic Phenotype in Immunized *Npc1^*nih*^* Mice

**Figure 4:**
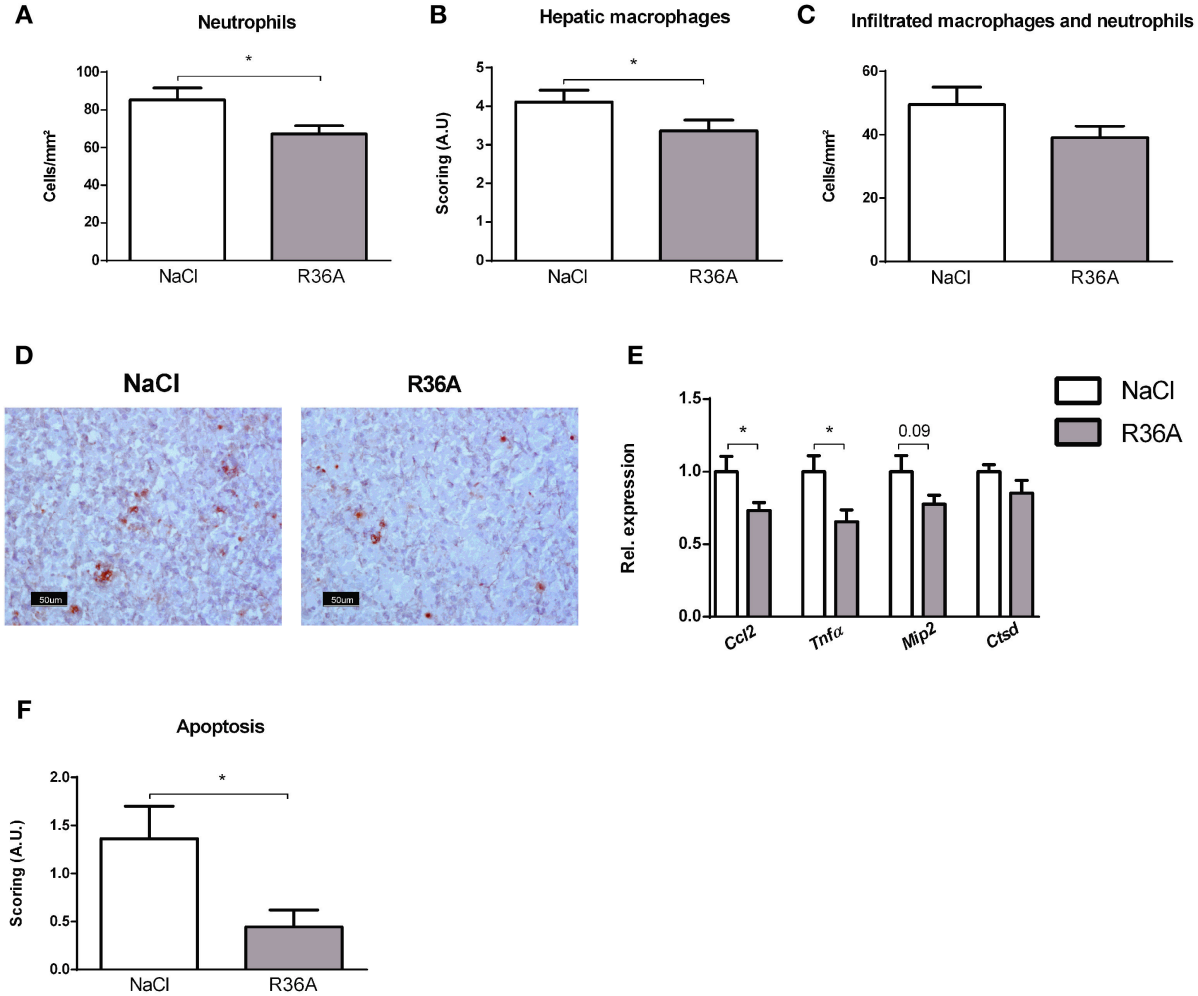
Hepatic parameters. **(A–D)** Liver sections were stained for neutrophils (NIMP), hepatic macrophages (CD68), and infiltrated macrophages and neutrophils (Mac-1). NIMP and Mac-1 positive cells were counted, whereas the CD68 stainings were scored. Panel D shows representative images of the NIMP staining. **(E)** Hepatic gene expression analysis of inflammatory markers *Ccl2, Tnf*α, and *Mip2*. Data are shown relative to NaCl-treated *Npc1*^*nih*^ mice. **(F)** Quantification of hepatic apoptosis, assessed by scoring of HE-staining of the liver. ^*^Indicates *p* < 0.05 compared to NaCl-treated *Npc1*^*nih*^ mice by use of two-tailed unpaired *t-*test. *n* = 11–12 mice per group. All error bars are SEM.

**Figure 5:**
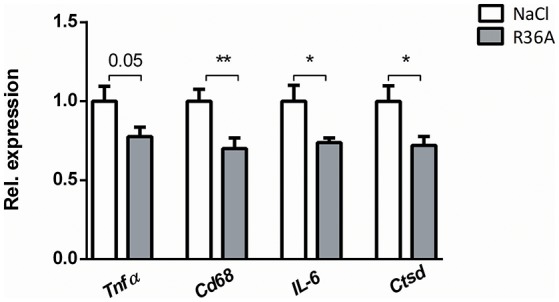
Parameters of spleen inflammation. Hepatic gene expression analysis of *Tnf*α*, Cd68, IL-6*, and *Ctsd*. Data are shown relative to NaCl-treated *Npc1*^*nih*^ mice. ^*^Indicates *p* < 0.05 and ^**^p < 0.01 compared to NaCl-treated *Npc1*^*nih*^ mice by use of two-tailed unpaired *t-*test. *n* = 11–12 mice per group. All error bars are SEM.

To determine whether immunization of *Npc1*^*nih*^ mice affects hepatic inflammation, hepatic cryosections were stained for the inflammatory cell markers NIMP (neutrophils), CD68 (resident macrophages) and Mac-1 (infiltrated macrophages and neutrophils). The number of infiltrated neutrophils and macrophages in control-treated *Npc1*^*nih*^ mice was increased compared to wildtype mice, confirming increased hepatic inflammation in *Npc1*^*nih*^ mice (Supplementary Table S1). Following pneumococcal immunization, the number of neutrophils and resident macrophages were reduced *Npc1*^*nih*^ mice (Figures 4A,B,D). Although the number of infiltrated neutrophils and macrophages (Figure 4C) and HE-scoring of hepatic inflammation (Figure 3C and Supplementary Figure S6) did not reach statistical significance between study groups, a trend toward reduction of these parameters following immunization was observed. To confirm these histological findings, hepatic gene expression analysis was performed on the inflammatory markers tumor necrosis factor alpha (*Tnf*α), chemokine (C-C motif) ligand 2 (*Ccl2*), macrophage inflammatory protein 2 (*Mip2*). *Tnf*α and *Ccl2* expression levels were significantly reduced in immunized *Npc1*^*nih*^ mice compared to non-immunized *Npc1*^*nih*^ mice (Figure 4E). The expression of *Mip2* and *Ctsd*, a marker for disease severity, showed no difference between control-treated and immunized *Npc1*^*nih*^ mice, although a trend suggesting reduced expression following immunization was observed (Figure 4E). As increased hepatic apoptosis is linked to inflammation and damage of the liver (41), hepatic apoptosis was also assessed. The amount of apoptotic cells, as quantified by HE-staining, was reduced upon immunization (Figure 4F), indicating an improvement of the NPC1 disease phenotype in the liver after pneumococcal immunization. Additionally, similar results were observed in the spleen, showing reduced expression of inflammatory markers, although in the case of *Tnf*α, only a trend was observed (Figure 5). Finally, hepatosplenic expression of most of these markers correlated with the plasma anti-oxLDL IgM autoantibody levels (Supplementary Figure S4), underlining the link between the systemic improvements and the anti-oxLDL IgM autoantibody levels. Altogether, these findings indicate an improved hepatosplenic phenotype in *Npc1*^*nih*^ mice that received the heat-inactivated pneumococci immunization, supporting its potential as treatment for NPC1 disease.

## Discussion

Due to severity of symptoms, delayed diagnosis and restricted therapeutical options, NPC1 disease patients suffer from a gradual impairment in quality of life and premature death. NPC1 disease commonly manifests through systemic symptoms, such as liver and spleen dysfunction, which precede neurological symptoms (epilepsy, movement disorder, dementia, among others). In this study, we show that heat-inactivated pneumococci immunization improves both systemic and neurological symptoms, including motor function, neurodegeneration, neuro- and systemic inflammation in a mouse model of NPC1 disease. In light of these promising results, we propose pneumococcal immunization as a novel therapeutic tool to ameliorate NPC1 disease severity, potentially in combination with treatments such as Miglustat and cyclodextrin (9, 42).

Although the role of oxLDL in neurodegenerative diseases has not been extensively investigated, oxLDL is involved in the pathogenesis of NASH and atherosclerosis, two diseases that share pathological mechanisms with NPC1 disease (19, 20, 43–46). Currently, there is no definite consensus on the benefits of targeting oxLDL by increasing anti-oxLDL antibodies in atherosclerosis, as some studies suggest that this approach increases inflammation and exacerbates disease severity (47–49). However, it should be noted that, in these studies, no distinction was made between classes and idiotypes of anti-oxLDL antibodies. Although a study from Smook et al (50) found no correlation between anti-oxLDL IgM autoantibody levels and advanced atherosclerotic lesion formation, this study could not establish whether anti-oxLDL IgM antibodies are anti or pro-atherogenic, and several other *in vivo* reports have found increased anti-oxLDL IgM antibody levels to be beneficial (51, 52). Thus, it is likely that the reported pathogenic effects of anti-oxLDL antibodies are due to the production of anti-oxLDL IgG, rather than IgM, antibodies. In atherosclerosis and NASH, pneumococcal immunization has been shown to ameliorate inflammation and disease severity (18, 20, 31), suggesting that this treatment may be beneficial to the systemic components of NPC1.

Although no effects were observed on splenic lipid accumulation following pneumococcal immunization, expression of several inflammatory genes was improved in the spleen of immunized *Npc1*^*nih*^ mice. Of note, in this study, we observed reduced spleen weight in control-treated *Npc1*^*nih*^ mice compared to wildtype mice instead of characteristic splenomegaly of NPC1 disease, indicating that this model does not recapitulate the spleen NPC1 disease phenotype. In contrast to spleen observations, liver weight and cholesterol accumulation as well as hepatic inflammation were increased in control-treated *Npc1*^*nih*^ mice compared to wildtype counterparts. Following pneumococcal immunization, liver apoptosis, hepatosplenic inflammation and hepatic cholesterol accumulation were decreased in immunized *Npc1*^*nih*^ mice, although the latter effects were of low magnitude. In addition to targeting oxLDL, immunization with heat-inactivated pneumococci is also known to improve clearance of apoptotic cells, due to molecular mimicry between apoptotic cells and *S. pneumoniae* (31). Indeed, after immunization, *Npc1*^*nih*^ mice displayed lower levels of apoptotic liver cells. Therefore, the beneficial effects of heat-inactivated pneumococci immunization in our study can partly derive from increased clearance of apoptotic cells.

As expected, control-treated *Npc1*^*nih*^ mice showed motor function deficits as assessed by the pole and rotarod tests, confirming neurological impairments in these mice compared to wildtype mice. In this study, unlike control-treated *Npc1*^*nih*^ mice, the performance of immunized *Npc1*^*nih*^ mice on the pole test remained unchanged over time. Additionally, immunized *Npc1*^*nih*^ mice displayed less prominent decay on rotarod test performance compared to their control-treated counterparts. It should be noted that, unlike the beam walking or the catwalk test, several neuronal circuits are involved in pole and rotarod test performance (53). As such, we are unable to pinpoint the specific skills that were improved by pneumococcal immunization. Nonetheless, our results indicate an overall improvement in motor function decay following pneumococcal immunization in *Npc1*^*nih*^ mice, in line with observed reduction in neuroinflammation and cerebellar damage.

The mechanisms leading to improved neurological features in *Npc1*^*nih*^ mice following pneumococcal immunization are unclear. IgM antibodies are large molecules and are thus usually precluded from the nervous system due to the blood brain barrier (BBB). As such, whether peripheral immunization strategies can be effective in the nervous system is a controversial matter. Nonetheless, peripheral immunization in Alzheimer's disease and amyotrophic lateral sclerosis has been shown to elicit beneficial effects in the nervous system with as little as 0.1% antibody passage through the BBB (54, 55). Furthermore, it has been proposed that antibodies in the nervous system facilitate the corresponding ligand efflux to the periphery, thus contributing to its clearance (55). As such, it is possible that the neurological improvements observed in this study derive from anti-oxLDL IgM autoantibody-mediated oxLDL clearance to the periphery and/or prevention of oxLDL uptake by microglia scavenger receptors, as has been proposed for macrophages (56, 57). On the other hand, live *S. pneumoniae* can cross the BBB due to certain membrane-surface proteins (58) that may be retained after heat-inactivating the bacteria. Although more thorough analyses are required to derive conclusions regarding microglia activation state, following pneumococcal immunization we observed a shift in microglia morphology that is commonly associated with a phenotypical change in these cells (59). While we are unable to pinpoint whether this is a response to interaction of microglia with anti-oxLDL IgM autoantibodies, complement activation or heat-inactivated pneumococci recognition, the latter option is unlikely, as previous reports suggest that heat-inactivated *S. pneumoniae* are non-immunogenic in the nervous system (60). Of note, *in vitro* and *in vivo* studies have shown that endothelial cells in the BBB are susceptible to oxLDL-induced cytotoxicity (61–63). Therefore, it is possible that high levels of plasma oxidized cholesterol products and oxLDL in NPC1 disease increase BBB permeability and exacerbate neurological symptoms, a concept that has previously been proposed in Alzheimer's disease (63). In support of these observations, the observed immune cells in the cortex of untreated *Npc1*^*nih*^ mice may result from a compromised BBB integrity. As such, improved neurologic symptoms following immunization in NPC1 disease could further derive from an improved BBB phenotype and function.

Persistent systemic- and neuroinflammation are well-described characteristics of NPC1 disease. While, under physiological conditions, inflammatory processes encourage the clearance of toxic compounds and the repair of injured tissues, the inability to overcome a pathological stimulus causes the persistent inflammatory response to become detrimental. Therefore, reducing inflammation and its associated pathological burden might be a promising approach to reduce NPC1 disease severity. In line with this view, administering non-steroidal anti-inflammatory drugs (NSAID) has been shown to improve motor function, body weight and even survival in a mouse model for NPC1 disease (64). Based on these findings, one can speculate on the direct role of inflammation in NPC1 disease severity and progression, despite it not being the primary cause of the disease. Similarly, though also initially not acknowledged as an essential disease component, inflammation is now accepted as a key component in disorders such as atherosclerosis (65), and Alzheimer's disease (66). Therefore, the role of inflammation in NPC1 disease and its usefulness as a therapeutical target should be further investigated.

Currently, therapeutic interventions for NPC1 disease focus on symptom management, and no curative treatments are available (67). As several mechanisms downstream of lysosomal lipid accumulation contribute to NPC1 disease pathology, interventions targeting different key features of NPC1 disease are likely to have increased benefits compared to mono-therapeutic startegies. In line with this rationale, *in vitro* and *in vivo* studies have analyzed the effects of combining therapies to improve cellular lipid accumulation, oxidative stress, inflammation and intracellular calcium homeostasis (64, 68). Overall, such studies found that combined therapies outperform the use of single compounds, highlighting the benefits of tackling different pathological mechanisms in NPC1 disease. For instance, the effects of improved cellular lipid accumulation, considered to be at the root of NPC1 disease pathology, are amplified when combined with anti-inflammatory and antioxidant compounds (68). As such, the investigation of pneumococcal immunization in combination with direct lysosomal lipid mobilizers (i.e., cyclodextrin) may be an attractive future approach for NPC1 disease patients.

Despite the promising features of pneumococcal vaccination, some points should be addressed before it can be used as a treatment for NPC1 disease. Although the impact of spleen damage on the immune system of NPC1 disease patients has yet to be analyzed, it is likely that the latter is compromised to some extent, which may in turn decrease the efficacy of immunotherapy. Furthermore, it was not yet assessed whether pneumococcal immunization is efficient in the context of NPC1 disease not just as a treatment but also as a preventive tool. In addition, in this study, *Npc1*^*nih*^ mice received two booster pneumococci immunizations, whereas pneumococcal vaccination in humans is usually performed once every several years, which may not be enough for therapeutically relevant oxLDL clearance (69). Nevertheless, it is worth taking into consideration that one of the most common manifestations of NPC1 is dysphagia, whose complications range from malnutrition to lung infections, mainly, bronchopneumonia (70–73). The fact that bronchopneumonia is a major cause of death in NPC1 disease patients is therefore another compelling argument for using pneumococcal immunization in these patients. The use of pneumococcal vaccinations against pneumonia is widespread (74–78). Furthermore, due to its long practice in the clinic, many optimizations of the vaccine have taken place, guaranteeing its safety even in vulnerable individuals such as pregnant women, young children, and the elderly (75–77). It should be noted that different S. *pneumoniae* strains as well as other bacteria, possess the phosphorylcholine epitope that triggers the production of anti-oxLDL IgM autoantibodies by means of molecular mimicry (31, 79). Nevertheless, it was so far not investigated whether immunization using these bacteria also elicits beneficial effects on lipid metabolism and inflammation. Furthermore, in this study, some of the phenotypical improvements, although statistically significant, were of low magnitude (i.e., hepatic cholesterol accumulation). Whether these effects can be enhanced by combining pneumococcal immunization with other NPC1 disease treatments or by using different immunogens should be explored in the future. Overall, considering the promising results in this study, further research is warranted to fully assess the most efficient way to modulate anti-oxLDL immune responses via pneumococcal vaccination in NPC1 disease patients.

## Final remarks

For the first time, we demonstrate that increasing anti-oxLDL IgM autoantibodies ameliorates motor function decay, neuro- and hepatic inflammation as well as hepatic cholesterol metabolism in NPC1 disease. In light of the present findings, we propose pneumococcal immunization, potentially in combination with other available treatments, as a novel therapeutic tool for NPC1 disease.

## Author Contributions

THo, IM, CB, JP, RS-S did the conception and design of the study. THo, IM, THe, HS, MG were involved with acquisition of data. THo, IM, THe, DC, JP, RS-S performed the (statistical) analysis and interpretation of data. THo, IM, DC, CB, JP, RS-S drafted the manuscript. THo, IM, YO, MW, and RS-S revised the manuscript and data. All authors were involved in revising the paper critically and gave final approval of the version to be submitted.

### Conflict of Interest Statement

The authors declare that the research was conducted in the absence of any commercial or financial relationships that could be construed as a potential conflict of interest.

## Supplementary Material

Supplementary Figure S1 Overview of experimental set-up.

Supplementary Figure S2 Representative pictures of microglia phenotype scoring (400x).

Supplementary Figure S3 General parameters. **(A)** Weight of *Npc1*^*nih*^ mice throughout study. **(B,C)** Absolute liver and spleen weight.

Supplementary Figure S4 Correlations between IgM antibody levels and gene expression levels of markers in the brain, liver, and spleen.

Supplementary Figure S5 Representative pictures and scoring of calbindin staining of Purkinje cells in cerebellum (400x) **(A)**. General histology of the brain by HE staining: Purkinje cells (top panel, 400x) and immune cell (bottom left panel, 200x) **(B)**.

Supplementary Figure S6 Liver HE inflammation scoring.

Supplementary Table S1 Parameters of 7 week-old wildtype and control-treated *Npc1*^*nih*^ mice.

## Abbreviations

NPC1: Niemann-Pick type C1
NASH: non-alcoholic steatohepatitis
oxLDL: oxidized low-density lipoprotein
PC: phosphorylcholine
T_LA_: locomotor activity time
HE: hematoxylin and eosin
Cxcl10: C-X-C motif chemokine 10
Ccl3: chemokine (C-C motif) ligand 3
Cd68: cluster of differentiation 68
Ctsd: cathepsin D
Acat2: acetyl-CoA acetyltransferase 2
Tnfα: tumor necrosis factor alpha
Ccl2: chemokine (C-C motif) ligand 2
Mip2: macrophage inflammatory protein 2
BBB: blood brain barrier.

## References

[1] Patterson MC Mengel E Wijburg FA Muller A Schwierin B Drevon H. Disease and patient characteristics in NP-C patients: findings from an international disease registry. Orphanet J Rare Dis. (2013) 8:12. 10.1186/1750-1172-8-12 23324478 PMC3558399

[2] Patterson MC Hendriksz CJ Walterfang M Sedel F Vanier MT Wijburg F. Recommendations for the diagnosis and management of Niemann-Pick disease type C: an update. Mol Genet Metab. (2012) 106:330–44. 10.1016/j.ymgme.2012.03.012 22572546

[3] Pfeffer SR. Clues to NPC1-mediated cholesterol export from lysosomes. Proc Natl Acad Sci USA. (2016) 113:7941–3. 10.1073/pnas.1608530113 27410046 PMC4961152

[4] Kelly DA Portmann B Mowat AP Sherlock S Lake BD. Niemann-Pick disease type C: diagnosis and outcome in children, with particular reference to liver disease. J Pediatr. (1993) 123:242–7. 10.1016/S0022-3476(05)81695-6 7688422

[5] Garver WS Francis GA Jelinek D Shepherd G Flynn J Castro G. The national niemann-Pick C1 disease database: report of clinical features and health problems. Am J Med Genet A (2007) 143A:1204–11. 10.1002/ajmg.a.31735 17497724

[6] Zhang H Wang Y Lin N Yang R Qiu W Han L. Diagnosis of Niemann-Pick disease type C with 7-ketocholesterol screening followed by NPC1/NPC2 gene mutation confirmation in Chinese patients. Orphanet J Rare Dis. (2014) 9:82. 10.1186/1750-1172-9-82 24915861 PMC4059728

[7] Greenberg CR Barnes JG Kogan S Seargeant LE. A rare case of Niemann-Pick disease type C without neurological involvement in a 66-year-old patient. Mol Genet Metab Rep. (2015) 3:18–20. 10.1016/j.ymgmr.2015.02.004 26937389 PMC4750631

[8] Sevin M Lesca G Baumann N Millat G Lyon-Caen O Vanier MT. The adult form of Niemann-Pick disease type C. Brain (2007) 130:120–33. 10.1093/brain/awl260 17003072

[9] Ory DS Ottinger EA Farhat NY King KA Jiang X Weissfeld L. Intrathecal 2-hydroxypropyl-beta-cyclodextrin decreases neurological disease progression in Niemann-Pick disease, type C1: a non-randomised, open-label, phase 1-2 trial. Lancet (2017) 390:1758–68. 10.1016/S0140-6736(17)31465-4 28803710 PMC6176479

[10] Alam MS Getz M Haldar K. Chronic administration of an HDAC inhibitor treats both neurological and systemic Niemann-Pick type C disease in a mouse model. Sci Transl Med. (2016) 8:326ra23. 10.1126/scitranslmed.aad9407 26888431

[11] Fu R Yanjanin NM Bianconi S Pavan WJ Porter FD. Oxidative stress in Niemann-Pick disease, type C. Mol Genet Metab. (2010) 101:214–8. 10.1016/j.ymgme.2010.06.018 20667755 PMC2950258

[12] Rimkunas VM Graham MJ Crooke RM Liscum L. TNF–α plays a role in hepatocyte apoptosis in Niemann-Pick type C liver disease. J Lipid Res. (2009) 50:327–33. 10.1194/jlr.M800415-JLR200 18815434 PMC2636917

[13] Alam MS Getz M Yi S Kurkewich J Safeukui I Haldar K. Plasma signature of neurological disease in the monogenetic disorder Niemann-Pick Type C. J Biol Chem. (2014) 289:8051–66. 10.1074/jbc.M113.526392 24488491 PMC3961638

[14] Sayre NL Rimkunas VM Graham MJ Crooke RM Liscum L. Recovery from liver disease in a Niemann-Pick type C mouse model. J Lipid Res. (2010) 51:2372–83. 10.1194/jlr.M007211 20418540 PMC2903820

[15] Tabas I Garcia-Cardena G Owens GK. Recent insights into the cellular biology of atherosclerosis. J Cell Biol. (2015) 209:13–22. 10.1083/jcb.201412052 25869663 PMC4395483

[16] Musso G Cassader M Gambino R. Non-alcoholic steatohepatitis: emerging molecular targets and therapeutic strategies. Nat Rev Drug Discov. (2016) 15:249–74. 10.1038/nrd.2015.3 26794269

[17] Hendrikx T Walenbergh SM Hofker MH Shiri-Sverdlov R. Lysosomal cholesterol accumulation: driver on the road to inflammation during atherosclerosis and non-alcoholic steatohepatitis. Obes Rev. (2014) 15:424–33. 10.1111/obr.12159 24629059

[18] Bieghs V van Gorp PJ Walenbergh SM Gijbels MJ Verheyen F Buurman WA. Specific immunization strategies against oxidized low-density lipoprotein: a novel way to reduce nonalcoholic steatohepatitis in mice. Hepatology (2012) 56:894–903. 10.1002/hep.25660 22334337 PMC3374908

[19] Houben T Oligschlaeger Y Bitorina AV Hendrikx T Walenbergh SMA Lenders M-H. Blood-derived macrophages prone to accumulate lysosomal lipids trigger oxLDL-dependent murine hepatic inflammation. Sci. Rep. (2017) 7:12550. 10.1038/s41598-017-13058-z 28970532 PMC5624963

[20] Jeurissen MLJ Walenbergh SMA Houben T Gijbels MJJ Li J Hendrikx T Prevention of oxLDL uptake leads to decreased atherosclerosis in hematopoietic NPC1-deficient Ldlr–/– mice. Atherosclerosis (2016) 255:59–65. 10.1016/j.atherosclerosis.2016.10.038 27816810

[21] Newman JW Morisseau C Hammock BD. Epoxide hydrolases: their roles and interactions with lipid metabolism. Prog Lipid Res. (2005) 44:1–51. 10.1016/j.plipres.2004.10.001 15748653

[22] Bjorkhem I Diczfalusy U. Oxysterols: friends, foes, or just fellow passengers? Arterioscler Thromb Vasc Biol. (2002) 22:734–42. 10.1161/01.ATV.0000013312.32196.49 12006384

[23] Porter FD Scherrer DE Lanier MH Langmade SJ Molugu V Gale SE. Cholesterol oxidation products are sensitive and specific blood-based biomarkers for Niemann-Pick C1 disease. Sci Transl Med. (2010) 2:56ra81. 10.1126/scitranslmed.3001417 21048217 PMC3170139

[24] Jiang X Sidhu R Porter FD Yanjanin NM Speak AO te Vruchte DT. A sensitive and specific LC-MS/MS method for rapid diagnosis of Niemann-Pick C1 disease from human plasma. J Lipid Res. (2011) 52:1435–45. 10.1194/jlr.D015735 21518695 PMC3122908

[25] Loftus SK Morris JA Carstea ED Gu JZ Cummings C Brown A. Murine model of Niemann-Pick C disease: mutation in a cholesterol homeostasis gene. Science (1997) 277(5323):232. 10.1126/science.277.5323.232 9211850

[26] Morris MD Bhuvaneswaran C Shio H Fowler S. Lysosome lipid storage disorder in NCTR-BALB/c mice I. Description of the disease and genetics. Am J Pathol. (1982) 108:140–9. 6765731 PMC1916074

[27] Shio H Fowler S Bhuvaneswaran C Morris MD. Lysosome lipid storage disorder in NCTR-BALB/c mice. II. Morphologic and cytochemical studies. Am J Pathol. (1982) 108:150–9. 6765732 PMC1916079

[28] Bhuvaneswaran C Morris MD Shio H Fowler S. Lysosome lipid storage disorder in NCTR-BALB/c mice. III. isolation and analysis of storage inclusions from liver. Am J Pathol. (1982) 108:160–70. 6101077 PMC1916082

[29] Reid PC Sugii S Chang T-Y. Trafficking defects in endogenously synthesized cholesterol in fibroblasts, macrophages, hepatocytes, and glial cells from Niemann-Pick type C1 mice. J Lipid Res. (2003) 44:1010–9. 10.1194/jlr.M300009-JLR200 12611909

[30] Hovakimyan M Maass F Petersen J Holzmann C Witt M Lukas J Combined therapy with cyclodextrin/allopregnanolone and miglustat improves motor but not cognitive functions in Niemann–Pick Type C1 mice. Neuroscience (2013) 252:201–11. 10.1016/j.neuroscience.2013.08.001 23948640

[31] Binder CJ Horkko S Dewan A Chang MK Kieu EP Goodyear CS. Pneumococcal vaccination decreases atherosclerotic lesion formation: molecular mimicry between *Streptococcus pneumoniae* and oxidized LDL. Nat Med. (2003) 9:736–43. 10.1038/nm876 12740573

[32] Briles DE Forman C Hudak S Claflin JL. Anti-phosphorylcholine antibodies of the T15 idiotype are optimally protective against *Streptococcus pneumoniae*. J Exp Med. (1982) 156:1177–85. 10.1084/jem.156.4.1177 7153709 PMC2186814

[33] Bieghs V Wouters K van Gorp PJ Gijbels MJ de Winther MP Binder CJ. Role of scavenger receptor A and CD36 in diet-induced nonalcoholic steatohepatitis in hyperlipidemic mice. Gastroenterology (2010) 138:2477–86, 86.e1–3. 10.1053/j.gastro.2010.02.051 20206177 PMC3114629

[34] Bieghs V Verheyen F van Gorp PJ Hendrikx T Wouters K Lutjohann D. Internalization of modified lipids by CD36 and SR-A leads to hepatic inflammation and lysosomal cholesterol storage in Kupffer cells. PLoS ONE (2012) 7:e34378. 10.1371/journal.pone.0034378 22470565 PMC3314620

[35] Pelsers MM Butler PJ Bishop CM Glatz JF. Fatty acid binding protein in heart and skeletal muscles of the migratory barnacle goose throughout development. Am J Physiol. (1999) 276:R637–43. 10.1152/ajpregu.1999.276.3.R637 10070122

[36] Loncarevic-Vasiljkovic N Pesic V Todorovic S Popic J Smiljanic K Milanovic D. Caloric restriction suppresses microglial activation and prevents neuroapoptosis following cortical injury in rats. PLOS ONE (2012) 7:e37215. 10.1371/journal.pone.0037215 22615943 PMC3352891

[37] Ogawa N Hirose Y Ohara S Ono T Watanabe Y. A simple quantitative bradykinesia test in MPTP-treated mice. Res Commun Chem Pathol Pharmacol. (1985) 50:435–41. 3878557

[38] Matsuura K Kabuto H Makino H Ogawa N. Pole test is a useful method for evaluating the mouse movement disorder caused by striatal dopamine depletion. J Neurosci Methods (1997) 73:45–8. 10.1016/S0165-0270(96)02211-X 9130677

[39] German DC Quintero EM Liang C Xie C Dietschy JM. Degeneration of neurons and glia in the Niemann-Pick C mouse is unrelated to the low-density lipoprotein receptor. Neuroscience (2001) 105:999–1005. 10.1016/S0306-4522(01)00230-5 11530237

[40] Marques AR Gabriel TL Aten J van Roomen CP Ottenhoff R Claessen N. Gpnmb is a potential marker for the visceral pathology in Niemann-Pick type C disease. PLoS ONE (2016) 11:e0147208. 10.1371/journal.pone.0147208 26771826 PMC4714856

[41] Zhan SS Jiang JX Wu J Halsted C Friedman SL Zern MA. Phagocytosis of apoptotic bodies by hepatic stellate cells induces NADPH oxidase and is associated with liver fibrosis *in vivo*. Hepatology (2006) 43:435–43. 10.1002/hep.21093 16496318

[42] Wraith JE Imrie J New therapies in the management of Niemann-Pick type C disease: clinical utility of miglustat. Therapeutics and clinical risk management. (2009) 5:877–87. 10.2147/TCRM.S5777 PMC2781062 19956552

[43] Hörkkö S Binder CJ Shaw PX Chang M-K Silverman G Palinski W. Immunological responses to oxidized LDL. Free Rad Biol Med. (2000) 28:1771–9. 10.1016/S0891-5849(00)00333-6 10946219

[44] Colles SM Maxson JM Carlson SG Chisolm GM. Oxidized LDL-induced injury and apoptosis in atherosclerosis: potential roles for oxysterols. Trends Cardiovasc Med. (2001) 11:131–8. 10.1016/S1050-1738(01)00106-2 11686002

[45] Praticò D. Lipid peroxidation in mouse models of atherosclerosis. Trends Cardiovasc Med. (2001) 11:112–6. 10.1016/S1050-1738(01)00099-8 11685999

[46] Palinski W Witztum JL. Immune responses to oxidative neoepitopes on LDL and phospholipids modulate the development of atherosclerosis. J Internal Med. (2001) 247:371–80. 10.1046/j.1365-2796.2000.00656.x 10762454

[47] Shoenfeld Y Wu R Dearing Linda D Matsuura E Are anti–oxidized low-density lipoprotein antibodies pathogenic or protective? Circulation (2004) 110:2552–8. 10.1161/01.CIR.0000143225.07377.EA 15505108

[48] Zhang J Wang D He S. Roles of antibody against oxygenized low density lipoprotein in atherosclerosis: recent advances. Int J Clin Exp Med. (2015) 8:11922–9. 26550105 PMC4612790

[49] Lopes-Virella MF Virella G. Clinical significance of the humoral immune response to modified LDL. Clin Immunol. (2010) 134:55–65. 10.1016/j.clim.2009.04.001 19427818 PMC2808452

[50] Smook MLF van Leeuwen M Heeringa P Damoiseaux JGMC Theunissen R Daemen MJAP. Anti-oxLDL antibody isotype levels, as potential markers for progressive atherosclerosis in APOE and APOECD40L mice. Clin Exp Immunol. (2008) 154:264–9. 10.1111/j.1365-2249.2008.03746.x 18778362 PMC2612714

[51] Hosseini H Li Y Kanellakis P Tay C Cao A Tipping P. Phosphatidylserine liposomes mimic apoptotic cells to attenuate atherosclerosis by expanding polyreactive IgM producing B1a lymphocytes. Cardiovasc Res. (2015) 106:443–52. 10.1093/cvr/cvv037 25681396

[52] Shaw PX Hörkkö S Chang M-K Curtiss LK Palinski W Silverman GJ. Natural antibodies with the T15 idiotype may act in atherosclerosis, apoptotic clearance, and protective immunity. J Clin Investig. (2000) 105:1731–40. 10.1172/JCI8472 10862788 PMC378505

[53] Mann A Chesselet M-F Chapter 8 - Techniques for Motor Assessment in Rodents. In: LeDoux MS, editor. Movement Disorders. 2nd edn. Boston: Academic Press (2015). p. 139–57. 10.1016/B978-0-12-405195-9.00008-1

[54] Banks WA Terrell B Farr SA Robinson SM Nonaka N Morley JE. Passage of amyloid β protein antibody across the blood–brain barrier in a mouse model of Alzheimer's disease. Peptides (2002) 23:2223–6. 10.1016/S0196-9781(02)00261-9 12535702

[55] Xu X Denic A Jordan LR Wittenberg NJ Warrington AE Wootla B. A natural human IgM that binds to gangliosides is therapeutic in murine models of amyotrophic lateral sclerosis. Dis Models Mech. (2015) 8:831. 10.1242/dmm.020727 26035393 PMC4527295

[56] Wilkinson K El Khoury J. Microglial scavenger receptors and their roles in the pathogenesis of Alzheimer's disease. Int J Alzheimer Dis. (2012) 2012:489456. 10.1155/2012/489456 22666621 PMC3362056

[57] Hörkkö S Bird DA Miller E Itabe H Leitinger N Subbanagounder G. Monoclonal autoantibodies specific for oxidized phospholipids or oxidized phospholipid-protein adducts inhibit macrophage uptake of oxidized low-density lipoproteins. J Clin Investig. (1999) 103:117–28. 9884341 10.1172/JCI4533 PMC407862

[58] Coureuil M Lécuyer H Bourdoulous S Nassif X. A journey into the brain: insight into how bacterial pathogens cross blood–brain barriers. Nat Rev Microbiol. (2017) 15:149–59. 10.1038/nrmicro.2016.178 28090076

[59] Fernández-Arjona MdM Grondona JM Granados-Durán P Fernández-Llebrez P López-Ávalos MD. Microglia morphological categorization in a rat model of neuroinflammation by hierarchical cluster and principal components analysis. Front Cell Neurosci. (2017) 11:235. 10.3389/fncel.2017.00235 28848398 PMC5550745

[60] Mook-Kanamori BB Geldhoff M van der Poll T van de Beek D. Pathogenesis and pathophysiology of pneumococcal meningitis. Clin Microbiol Rev. (2011) 24:557–91. 10.1128/CMR.00008-11 21734248 PMC3131058

[61] Lin Y-L Chang H-C Chen T-L Chang J-H Chiu W-T Lin J-W. Resveratrol protects against oxidized LDL-induced breakage of the blood-brain barrier by lessening disruption of tight junctions and apoptotic insults to mouse cerebrovascular endothelial cells. J Nutr. (2010) 140:2187–92. 10.3945/jn.110.123505 20980646

[62] Schreurs MPH Cipolla MJ. Cerebrovascular dysfunction and blood-brain barrier permeability induced by oxidized LDL are prevented by apocynin and magnesium sulfate in female rats. J Cardiovasc Pharmacol. (2014) 63:33–9. 10.1097/FJC.0000000000000021 24084218 PMC3909873

[63] Dias Irundika HK Polidori Maria C Griffiths Helen R. Hypercholesterolaemia-induced oxidative stress at the blood–brain barrier. Biochem Soc Trans. (2014) 42:1001–5. 10.1042/BST20140164 25109993

[64] Smith D Wallom K-L Williams IM Jeyakumar M Platt FM. Beneficial effects of anti-inflammatory therapy in a mouse model of Niemann-Pick disease type C1. Neurobiol Dis. (2009) 36:242–51. 10.1016/j.nbd.2009.07.010 19632328

[65] Libby P. Inflammation in Atherosclerosis. Arterioscl Thromb Vasc Biol. (2012) 32:2045–51. 10.1161/ATVBAHA.108.179705 22895665 PMC3422754

[66] McGeer PL McGeer EG. The amyloid cascade-inflammatory hypothesis of Alzheimer disease: implications for therapy. Acta Neuropathol. (2013) 126:479–97. 10.1007/s00401-013-1177-7 24052108

[67] Madra M Sturley SL. Niemann–Pick type C pathogenesis and treatment: from statins to sugars. Clin Lipidol. (2010) 5:387–95. 10.2217/clp.10.19 21394236 PMC3050622

[68] Williams IM Wallom K-L Smith DA Al Eisa N Smith C Platt FM. Improved neuroprotection using miglustat, curcumin and ibuprofen as a triple combination therapy in Niemann–Pick disease type C1 mice. Neurobiol Dis. (2014) 67:9–17. 10.1016/j.nbd.2014.03.001 24631719

[69] Hurley LP Allison MA Pilishvili T O'Leary ST Crane LA Brtnikova M. Primary care physicians' struggle with current adult pneumococcal vaccine recommendations. J Am Board Family Med. (2018) 31:94–104. 10.3122/jabfm.2018.01.170216 29330244 PMC5774021

[70] Wraith JE Guffon N Rohrbach M Hwu WL Korenke GC Bembi B. Natural history of Niemann-Pick disease type C in a multicentre observational retrospective cohort study. Mol Genet Metabol. (2009) 98:250–4. 10.1016/j.ymgme.2009.06.009 19616462

[71] Iturriaga C Pineda M Fernández-Valero EM Vanier MT Coll MJ. (2013). Niemann-Pick C disease in spain: clinical spectrum and development of a disability scale. J Neurol Sci. (2013) 249:1–6. 10.1016/j.jns.2006.05.054 16814322

[72] Wraith JE Baumgartner MR Bembi B Covanis A Levade T Mengel E. Recommendations on the diagnosis and management of Niemann-Pick disease type C. Mol Genet Metab. (2009) 98:152–65. 10.1016/j.ymgme.2009.06.008 19647672

[73] Walterfang M Chien Y-H Imrie J Rushton D Schubiger D Patterson MC. Dysphagia as a risk factor for mortality in Niemann-Pick disease type C: systematic literature review and evidence from studies with miglustat. Orph J Rare Dis. (2012) 7:76. 10.1186/1750-1172-7-76 23039766 PMC3552828

[74] Jackson LA Gurtman A van Cleeff M Jansen KU Jayawardene D Devlin C. Immunogenicity and safety of a 13-valent pneumococcal conjugate vaccine compared to a 23-valent pneumococcal polysaccharide vaccine in pneumococcal vaccine-naive adults. Vaccine (2013) 31:3577–84. 10.1016/j.vaccine.2013.04.085 23688526

[75] Pomat WS van den Biggelaar AHJ Phuanukoonnon S Francis J Jacoby P Siba PM. Safety and immunogenicity of neonatal pneumococcal conjugate vaccination in papua new guinean children: a randomised controlled trial. PLOS ONE (2013) 8:e56698. 10.1371/journal.pone.0056698 23451070 PMC3579820

[76] Bryant KA Block SL Baker SA Gruber WC Scott DA. Safety and immunogenicity of a 13-valent pneumococcal conjugate vaccine. Pediatrics (2010) 125:866–75. 10.1542/peds.2009-1405 20435707

[77] Ciprero K Zykov KA Briko NI Shekar T Sterling TM Bitieva E. Safety and immunogenicity of a single dose 23-valent pneumococcal polysaccharide vaccine in Russian subjects. Hum Vaccines Immunother. (2016) 12:2142–7. 10.1080/21645515.2016.1165373 27149114 PMC4994758

[78] Namkoong H Funatsu Y Oishi K Akeda Y Hiraoka R Takeshita K. Comparison of the immunogenicity and safety of polysaccharide and protein-conjugated pneumococcal vaccines among the elderly aged 80 years or older in Japan: an open-labeled randomized study. Vaccine (2015) 33:327–32. 10.1016/j.vaccine.2014.11.023 25448102

[79] Kolberg J Høiby EA Jantzen E. Detection of the phosphorylcholine epitope in streptococci,haemophilus and pathogenicneisseriaeby immunoblotting. Microb Pathog. (1997) 22:321–9. 10.1006/mpat.1996.0114 9188087

