# Supplementary figures and images for: Pneumococcal Immunization Reduces Neurological and Hepatic Symptoms in a Mouse Model for Niemann-Pick Type C1 Disease
Source: Front Immunol. 2019 Jan 7;9:3089. doi: 10.3389/fimmu.2018.03089 (PMC6330339; doi:10.3389/fimmu.2018.03089)

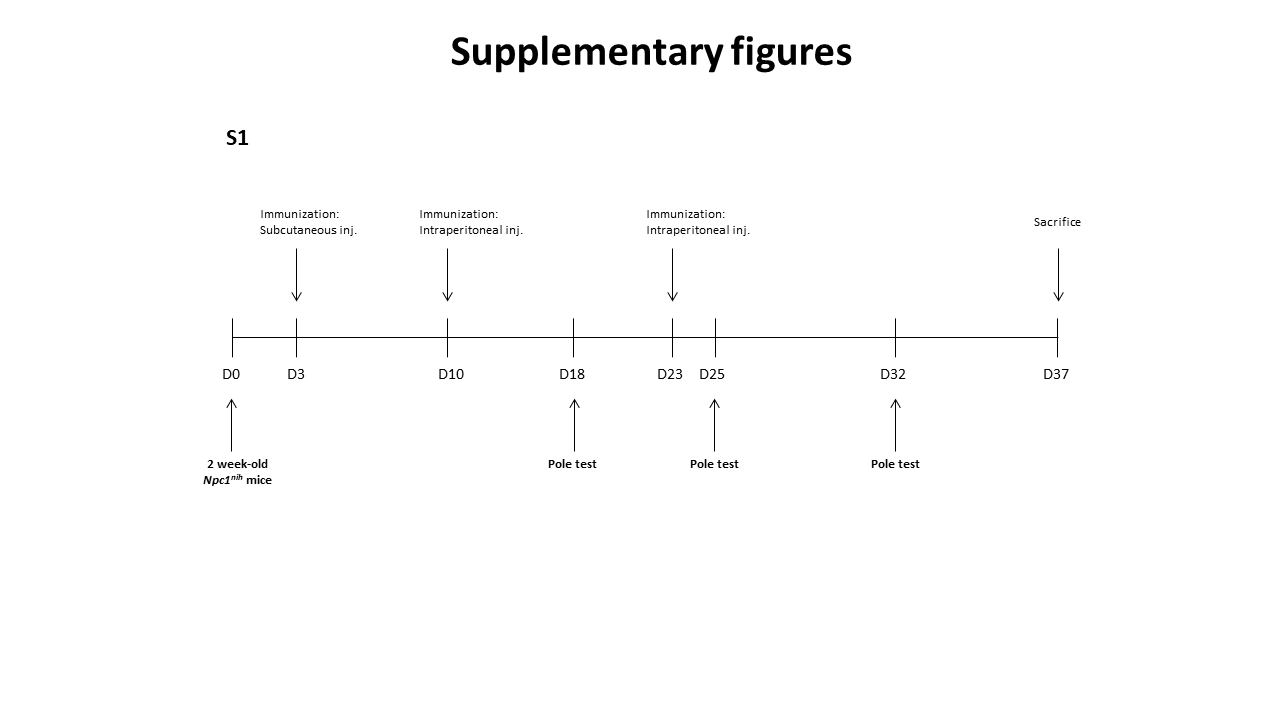

Supplement: Supplementary Figure S1 — Overview of experimental set-up. [file Image_1.tif]
