# Supplementary material for: Pneumococcal Immunization Reduces Neurological and Hepatic Symptoms in a Mouse Model for Niemann-Pick Type C1 Disease
Source: Front Immunol. 2019 Jan 7;9:3089. doi: 10.3389/fimmu.2018.03089 (PMC6330339; doi:10.3389/fimmu.2018.03089)
Supplement: Supplementary Table S1 — Parameters of 7 week-old wildtype and control-treated Npc1nih mice. [file Table_1.pdf]

Supplementary Table S1: parameters of 7 week-old wildtype and control-treated *Npc1^nih* mice

	Wt	*Npc1^nih*	*p value*
**Body parameters**			
Body weight	17.68 ± 0.56	13.69 ± 0.51	<0.0001
Liver weight	0.93 ± 0.04	1.20 ± 0.06	0.002
Spleen weight	0.17 ± 0.01	0.08 ± 0.01	<0.0001
**Motor function**			
Pole test			
t1	5.60 ± 0.36	9.28 ± 0.56	<0.0001
t2	5.89 ± 0.75	8.91 ± 0.53	0.003
t3	7.18 ± 0.76	13.96 ± 2.38	0.02
Rotarod			
t1	267.60 ± 10.83	231.0 ± 9.25	0.02
t2	245.7 ± 15.59	180.9 ± 10.94	0.003
**Histology**			
Infiltrating macrophages and neutrophils (Mac1) (# positive cells/mm^2)	29.74 ± 4.67	51.56 ± 5.64	0.01
**Cholesterol parameters**			
Plasma cholesterol (mM)	2.52 ± 0.11	2.96 ± 0.12	0.02
Liver cholesterol (mg total cholesterol/mg protein)	0.006 ± 0.0003	0.025 ± 0.0007	<0.0001

p value compares wildtype to *Npc1^nih* mice by use of two-tailed unpaired *t* test. Standards of error represent ± SEM.
